# Osteoblast Differentiation and Signaling: Established Concepts and Emerging Topics

**DOI:** 10.3390/ijms22136651

**Published:** 2021-06-22

**Authors:** Marco Ponzetti, Nadia Rucci

**Affiliations:** Department of Biotechnological and Applied Clinical Sciences, University of L’Aquila, Via Vetoio, Coppito 2, 67100 L’Aquila, Italy; marco.ponzetti@graduate.univaq.it

**Keywords:** osteoblast differentiation, extracellular vesicles, miRNAs, non-coding RNAs

## Abstract

Osteoblasts, the cells that build up our skeleton, are remarkably versatile and important cells that need tight regulation in all the phases of their differentiation to guarantee proper skeletal development and homeostasis. Although we know many of the key pathways involved in osteoblast differentiation and signaling, it is becoming clearer and clearer that this is just the tip of the iceberg, and we are constantly discovering novel concepts in osteoblast physiology. In this review, we discuss well-established pathways of osteoblastic differentiation, i.e., the classical ones committing mesenchymal stromal cells to osteoblast, and then osteocytes as well as recently emerged players. In particular, we discuss micro (mi)RNAs, long non-coding (lnc)RNAs, circular (circ)RNAs, and extracellular vesicles, focusing on the mechanisms through which osteoblasts are regulated by these factors, and conversely, how they use extracellular vesicles to communicate with the surrounding microenvironment.

## 1. Introduction

Bone is a very unique tissue. It partakes in many physiological functions, including movement, protection of soft organs, endocrine regulation of at least energy and phosphate metabolism [1], and it also acts as a reservoir of calcium and phosphate, which can be exploited at need [2]. Despite being mineralized and somewhat perceived as “immutable”, bone is actually very dynamic and active, especially during growth. In fact, the entire skeleton needs to be shaped to accommodate the growing organism in a mechanism that is termed bone modeling. If bone modeling fails, usually due to genetic diseases or severe malnutrition, the consequences are highly disabling at best, but more often fatal in infancy if not treated [3]. For bone modeling to succeed, bone needs to be continuously deposed and resorbed at the appropriate surfaces, in a coordinated and highly regulated fashion. This process involves mainly two cell types: osteoblasts, the bone-forming cells, and osteoclasts, the bone-resorbing cells, which are tightly controlled by environmental, hormonal, epigenetic, and mechanical cues. During bone modeling, osteoblasts have to produce more bone than what osteoclasts resorb since the organism is still growing, and therefore, the net result of bone modeling is bone accrual [4,5,6].

Once the bone mass peak is reached, the osteoblasts’ activity is counterbalanced by that of the osteoclasts, and therefore, the net result is a dynamic maintenance of the bone mass at a steady state, which is termed bone remodeling [7]. However, in time, due to only partially known factors, osteoblast activity starts losing ground versus osteoclastic bone resorption, and the result is age-induced osteopenia (literally “lack of bone”) or osteoporosis (literally “hole-ridden bone”) [8,9]. In women, the onset of menopause results in a drastic reduction in estrogen levels that causes net bone loss, which is further worsened by age-induced osteopenia/osteoporosis [10]. Understanding the mechanisms regulating all these physio-/pathological processes is a key research topic in bone biology and identifying new molecular players in these mechanisms could provide valuable therapeutic targets to prevent or revert bone diseases, such as osteoporosis, or even incurable genetic diseases.

In this review, we focus on one of the two key players in bone modeling/remodeling: the osteoblast, discussing the latest developments in the signaling and transcriptional regulation in osteoblast differentiation in physio-/pathological conditions.

## 2. From Mesenchymal Stem Cell to Osteoblast and Beyond: A Long Road

**Figure 1:**
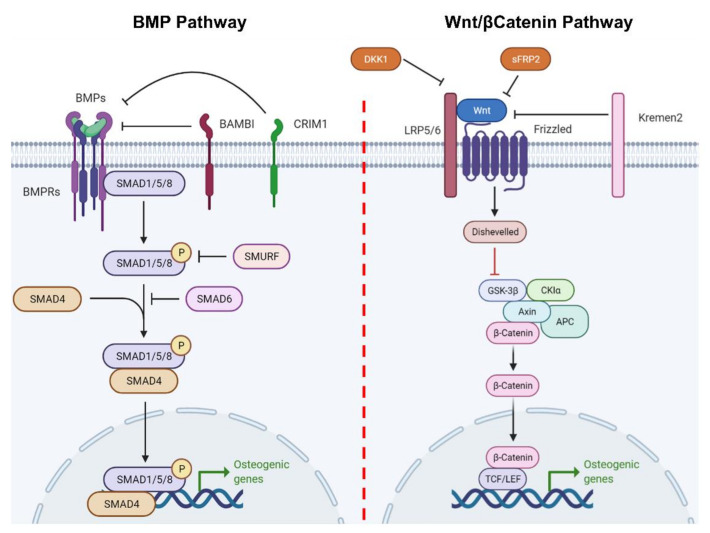
Conventional osteogenic differentiation pathways. Cartoon showing the two classical osteogenic differentiation pathways of MSCs/preosteoblasts, i.e., the bone morphogenic pathway and the Wnt/β-catenin pathway. BMPRs—BMP receptors; BAMBI—BMP and activin membrane-bound inhibitor homolog; CRIM1—cysteine-rich motor neuron 1 protein; SMAD—small mothers against decapentaplegic; DKK1—Dickkopf-related protein 1; sFRP2—secreted frizzled-related protein 2; Kremen—Kringle domain-containing transmembrane protein; LRP—Low Density Lipoprotein Receptor-Related Protein; GSK—glycogen synthase kinase; CKIα—casein kinase Iα; APC—adenomatous polyposis coli; TCF/LEF—T-cell factor/lymphoid enhancer-binding factor. Created with biorender.com.

**Figure 2:**
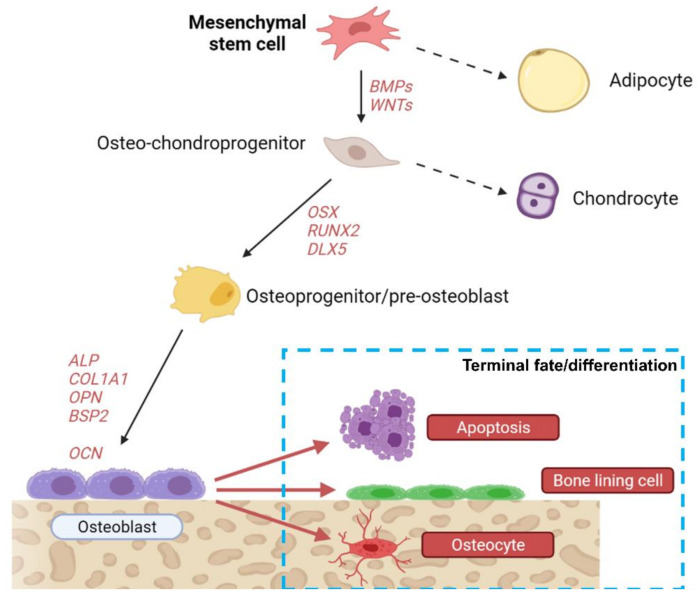
MSC differentiation. Cartoon showing the possible differentiation fates of mesenchymal stem cells. Created with biorender.com.

Osteoblasts derive from mesenchymal stem cells (MSCs) that reside in the bone marrow. These cells are multipotent since they can become osteoblasts, adipocytes, or chondrocytes, according to specific factors present in the microenvironment [11]. Indeed, their differentiation toward the osteogenic lineage is tightly controlled by different molecular factors, mainly belonging to the bone morphogenic proteins (BMPs) [12,13,14] and wingless-related integration site (WNT) pathways (Figure 1) [15,16,17,18,19], but involves many other pathways, including that of nuclear factor κB, which is typically considered important mostly for osteoclasts, as well as that of the NAD+-dependent deacetylase sirtuin-1. Osteoblast differentiation from MSCs also appears to be a feed-forward phenomenon as demonstrated by the fact that co-culturing MSCs with mature osteoblasts promotes MSC osteogenic differentiation [20,21]. Intriguingly, this feed-forward mechanism is at least partially mediated by soluble factors, and among these, extracellular vesicles may play an important role, as will be discussed in Section 3.2. The first step of differentiation is the commitment toward a common osteo-chondroprogenitor cell, which becomes further committed following the activation of master osteogenic transcriptional factors, such as runt-related transcription factor 2 (RUNX2), osterix (OSX) and drosophila distal-less 5 (DLX5). This committed osteoprogenitor cell then becomes a pre-osteoblast, following the transcription of early osteogenic genes, such as alkaline phosphatase (ALP) and collagen1α1 chain (COL1A1), the expression of which is then kept throughout the mature osteoblasts’ life, in addition to other common osteoblast markers, such as osteopontin (OPN), bone sialoprotein II (BSP II) and osteocalcin (OCN) [11]. Once osteoblasts finish their job of deposing bone where it is needed, they have three possible fates: (1) undergoing apoptosis; (2) becoming bone lining cells; or (3) becoming osteocytes (Figure 2) [22].

Bone lining cells are thought to protect the bone from osteoclastic resorption, and they are the key player in maintaining the “resting” phase of bone remodeling: in fact, osteoclasts must come into direct contact with bone surfaces for their final differentiation to happen, and bone lining cells represent a dynamic “passivating” layer that can give way to osteoclasts when resorption is needed, and vice versa [7]. The differentiation toward the osteocyte serves a different purpose and is most often observed during bone modeling. Osteocytes represent >90% of adult bone cells, and it is therefore unsurprising that their role in the bone tissue is crucial. They are mechanosensing cells, able to control bone remodeling and push it toward bone deposition or resorption based on mechanical cues [22]. Finally, they also have endocrinological functions, regulating the phosphate metabolism through fibroblast growth factor 23 (FGF23); more roles for osteocytes in cancer, and other pathologies are emerging constantly [23,24]. Apoptosis is probably the pathway that osteoblasts follow when neither of the two mentioned differentiation pathways are useful to bone homeostasis in that moment.

## 3. New Concepts in Osteoblast Differentiation Regulation

Although much is known about the classical pathways in osteoblasts determination and terminal differentiation, there are a number of novel ways through which this process is controlled and fine-tuned to match the continuously changing needs of the organism, whether it is in the growth or remodeling phase. An overview of these novel mechanisms is presented below.

### 3.1. Non-Coding RNAs

Non-coding RNA (ncRNA) is a generic term referring to any RNA that is not destined to being translated into a protein. Genes encoding for these kinds of RNAs reside in the non-exonic part of the genome, which is the overwhelming majority of the genetic material in complex organisms, including mice and humans. There are many different families of ncRNAs that have been discovered over the years, including transfer RNAs (tRNAs), micro RNAs (miRNAs), long non-coding RNAs (lncRNAs), piwi-interacting RNA (piRNAs), small nucleolar RNA (snoRNAs), small nuclear RNA (snRNAs), small Cajal body-specific RNA (scaRNAs) and the more-recently discovered circular RNAs (circRNAs) [25]. All of these have very different roles in cellular metabolism and are potentially important in osteoblasts, but for the purpose of this review, we focused on those which have been reported to be involved in osteoblast differentiation, namely miRNAs, lncRNAs and circRNAs.

#### 3.1.1. miRNAs in Osteoblast Differentiation

The class of ncRNA that is best characterized and that can pride itself with the highest number of studies overall is surely that of miRNAs (source: NIH Pubmed search). MiRNAs are 22 nt on average, and they canonically arise from the transcription of specific DNA sequences encoding for a longer primary miRNA (pri-miRNA) precursor, which is then processed into a precursor miRNA (pre-miRNA) that is finally processed into a mature miRNA that can exert its function through association with proteic effectors of the RNA-induced silencing complex (RISC) [26,27]. In general, miRNAs bind the 3′-UTR of mRNAs and mediate their silencing or degradation, although it has been reported that they can exert similar actions binding to other regions as well [26]. MiRNAs are extremely important for the normal development and homeostasis of virtually all cells and tissues; aberrant miRNA expression patterns are associated with severe diseases, such as cancer [28]. Many reports from the late 2000s to today showed that osteoblast differentiation also relies on miRNAs, and many of them were studied in bone physiopathology. As stated in the above paragraphs, Wnt and BMP are the most important pathways in osteoblast differentiation [11]. Several miRNAs are reported to target these two pathways, thus impairing or favoring osteoblast differentiation. The miR-29 family, namely miR-29a and miR-29b, is increased during osteoblastogenesis, thus promoting bone formation at early stages through silencing the key Wnt pathway inhibitors, DKK1, Kremen2, and sFRP2 [29]. Another cornerstone paper demonstrated that during BMP-2-induced osteogenic differentiation, miR-133 and miR-135 are downregulated, thus releasing the silencing of key pro-osteogenic factors, RUNX2 and SMAD5, the latter being a key molecule in BMP signaling [30]. Additionally, miR-20a is increased during osteogenic differentiation, and targets peroxisome proliferator activated receptor gamma (PPARγ), a negative regulator of BMP/RUNX2 signaling as well as BMP and activin membrane-bound inhibitor (Bambi) and cysteine-rich motor neuron 1 protein (Crim1), antagonists of the BMP pathway [31]. Two other important miRNAs that regulate the RUNX2 pathway are miR-2861 [32] and miR-3960 [33]. The first targets histone deacetylase 5 (HDAC5), which increases the proteasomal degradation of RUNX2, hence its suppression leads to an increased accumulation of RUNX2 [32]. A similar effect was identified for miR-3960, which targets the RUNX2 repressor Homeobox A2 (HOXA2) [33]. Intriguingly, miR-2861 and miR-3960 belong to the same miRNA cluster and are, therefore, co-expressed, creating an additive effect that strongly increases RUNX2-mediated osteogenesis [33]. Another indirect controller of RUNX2 is miR-15b, which targets SMAD Ubiquitination Regulatory Factor 1 (SMURF1), thus preventing RUNX2 ubiquitination [34]. Other miRNAs act as the other side of the coin by directly targeting RUNX2, thus determining a reduction in osteogenic differentiation, and therefore, are switched off during this process. A key example is miR-338-3p, which targets both RUNX2 and fibroblast growth factor receptor 2 (FGFR2) [35]. Similarly, miR-637 targets OSX, another master gene in osteoblast differentiation [36]. A completely different transcript, which, however, results in similar effects, is targeted by miR-138: focal adhesion kinase (FAK) [37]. This kinase is important in osteogenic differentiation, and its inhibition blunts this process severely [37,38]. As mentioned in the previous paragraph, osteoblasts can undergo apoptosis after they are finished deposing bone, and a significant inducer of this process is the lowering of estrogen levels in women [39]. This is reported to be partially accomplished through miR-17-92a upregulation by estrogen signaling [40]. Indeed, miRNA targets bcl-2-like protein 11 (BIM), a pro-apoptotic factor, hence preventing osteoblast cell death; conversely, estrogen deprivation (e.g., OVX and postmenopausal osteoporosis) leads to pro-apoptotic BIM upregulation [40]. Another recently studied miRNA in osteogenesis is miR-216a, promoting osteoblast differentiation and bone deposition by suppressing expression of Cbl proto-oncogene, which results in a block of the phosphoinositide 3-kinase (PI3K)/protein kinase B (PKB) pathway. The authors show that PI3K/PKB activation induces osteogenesis suppression following high-dose dexamethasone treatment, which is therefore, prevented by miR-216a [41]. Many other miRNAs are reported to have a role in osteogenesis [42]; however, their utility as biomarkers and therapeutic targets is currently lagging behind, probably due to the presence of well-established methods for the diagnosis of diseases such as osteoporosis, and the expensiveness of producing (along with the delivery difficulties) antagomiRs (synthetic antagonists of miRNAs) or miRNA mimics to be used in human therapy for bone diseases.

#### 3.1.2. lncRNAs in Osteoblast Differentiation

Long non-coding RNAs are, as the name suggests, longer than most other ncRNAs, being at least 200 nts and up to more than 10000 nts. They also share two key features with coding RNAs, i.e., a poly-A tail, and the 5′-cap [43]. Their considerable length allows lncRNAs to be versatile molecules, with reported functions spanning from control of mRNA transcription and translation, post-translational modifications, to definition of the chromatin structure; they can even give rise to small proteins and other smaller non-coding molecules, such as miRNAs, and act as miRNA “sponges”, i.e., they can provide binding sites for specific miRNAs, thus competitively inhibiting their binding to mRNA targets [44]. Although less studied than miRNAs, reports have emerged in the last few years suggesting roles for this class of ncRNAs in osteoblast differentiation. A novel lncRNA, identified by RNAseq and termed lncRNA-1, was found to increase osteoblastic differentiation, leading to the activation of several master regulators of osteogenesis, including RUNX2 and SP7 [44]. H19, one of the most abundant non-coding transcripts in mammals, is significantly upregulated during osteogenesis, from MSCs to mature osteoblasts [45]. The proposed mechanism of action is a miRNA sponge activity towards miR-22 and miR-141, both negative regulators of osteogenic differentiation [46], although its effect may be more complex, involving a feedback loop that also includes miR-675-5p [43], and also considering that the role of miR-22 in osteogenic differentiation is a matter of controversy, with another report stating that it is actually pro-osteogenic [47]. Anti-differentiation non-coding RNA (ANCR) is, instead, a negative determinant of osteogenic differentiation, which keeps MSCs in an undifferentiated state by physically interacting with the enhancer of zeste homolog 2 (EZH2) in the RUNX2 promoter regions, thus impairing its transcription [48]. A mirror mechanism of action was proposed for HOXA-AS3, which also acts through EZH2, but results in increased RUNX2 expression, which would explain its anti-adipogenic and pro-osteogenic effect on MSCs [49]. MIR31HG is another promoter of osteogenic differentiation, which is able to antagonize inflammation-induced osteogenic impairment through a direct interaction with nuclear factor of kappa light polypeptide gene enhancer in B-cells inhibitor, alpha (IκB-α) [50]. XR-111050 is another particularly interesting example, being able to increase osteogenic differentiation of MSCs through the upregulation of several key factors, including OCN, COL1A2, OPN, and RUNX2 [51]. The lncRNA HOTAIR is also noteworthy: its expression levels are reduced in BMP-2-induced osteogenic differentiation, and its overexpression reduces RUNX2 and COL1A1 expression, which is likely accomplished by transcriptional control of miR-17-5p promoter methylation, which causes its overexpression [52,53]. The field is relatively young and lncRNAs are particularly complex molecules to study; however, several findings related to their role in osteogenic differentiation and bone disease have been evidenced so far [54], and we expect the field to grow in the near future.

#### 3.1.3. circRNAs in Osteoblast Differentiation

CircRNAs, at variance with other ncRNAs, usually derive from alternative splicing of coding mRNAs, which come to be covalently linked at the 3′ and 5′ ends [55]. Intriguingly, their presence and expression levels seem to be independent of their linear, canonically-spliced isoforms, and they have a half-life of more than 48 h [55]. Although their function is still mostly obscure, they have been proposed to work as miRNA sponges, at least in some instances, and to function as splicing competitors for their linear cognate genes [56,57,58]. Although the mechanism of action is still unclear for most circRNAs, some of them show clear pro-osteogenic effects. An example is hsa-circ0074834, which is able to promote the healing of bone defects and stimulate osteogenic differentiation of bone marrow stromal cells (BMSCs) [59]. Recently, circRNAs minichromosome maintenance complex component 3 (circMCM3AP) and protein O-mannosyltransferase 1 (circPOMT1) were shown to promote osteogenesis through BMP signaling in a mechanism that seems to be dependent on RUNX2, COL1 and miR-6881-3p [60]. Another interesting example is circRNA 0076906, which seems to act as a miRNA sponge for miR-1305 in MSCs, resulting in an increased expression of osteoglycin (OGN) and leading to increased osteoblast differentiation [61].

### 3.2. Extracellular Vesicles

Extracellular vesicles (EVs) are nanoparticles surrounded by a phospholipid bilayer, with sizes that range from 20 to 1000 nm in diameter, and are naturally released by all cells [62]. EVs are emerging as important means of cell–cell communication, both locally and at a distance. EVs achieve this aim by shuttling a wide range of signaling molecules, including most of the ncRNAs discussed above, especially miRNAs, as well as mRNAs and specific membranes (e.g., tetraspanins) or intra-vesicular proteins. The fact that osteoblasts (and chondrocytes) secrete EVs has been known for more than 50 years, with the concept of matrix vesicles that enact bone mineralization through starting the nucleation of calcium phosphate minerals, as was first observed independently by Anderson and Bonucci in 1967, further investigated by Ornoy in the 1980s and recently reviewed by Hasegawa [63,64,65,66]. However, the idea that some of the EVs produced by osteoblasts can mediate cell–cell communication only emerged recently, and few reports are present in literature on the topic [67]. Matrix vesicles (MVs) are defined as 100–300 nm diameter lipid bilayer particles that can form apatite from calcium and phosphate ions in solution and bind to collagen fibrils [68]. These properties are due to a very specific intravesicular protein setup, composed of phosphatases, such as orphan phosphatase 1 (PHOSPHO1) and tissue non-specific alkaline phosphatase (TNAP), sphingomyelin phosphodiesterases 3 (SMPD3), ectonucleotide pyrophosphatase/phosphodiesterase 1 (ENPP1) and annexins that transport and bind calcium ions into the vesicular lumen [68]. All these proteins act in concert to start the nucleation of hydroxyapatite crystals and to bind collagen fibers in the extracellular matrix (ECM) so that the mineralization process can take place. However, the field underwent such a paradigm shift that Shapiro and colleagues proposed that matrix vesicles are a subgroup of extracellular vesicles that present only one important difference, i.e., that they are anchored to the ECM [69]: matrix vesicles are no longer the rule but an exception.

Building on this “updated” view of the topic, a first seminal paper identified osteoblast exosomes and analyzed their protein content, finding that they were mostly composed of membrane proteins resembling their cells of origin, enriched in components of the eukaryotic initiation factor (EIF)2 signaling pathway, and contained other potentially pro-osteogenic components, such as α5β1 integrin [70]. The content of EVs usually mirrors that of the cell of origin but they may also be enriched in specific molecules, which, in the case of osteoblasts, is strongly dependent on the differentiation status of the cell during mineralization, and the physiopathological *status* of the bone. In fact, the EVs content was shown to change during in vitro osteogenic differentiation with a predominance of matrix deposition-associated proteins in mineralizing osteoblasts [71], albeit most of the proteins contained in non-mineralizing osteoblasts (OB)-EVs being common with the mineralizing ones, while most divergences were found in matrix organization proteins, as well as in the vesicle size [71,72]. Another interesting report showed that BMSCs-derived EVs harvested from osteoporotic/osteoarthritic patients reduced osteogenesis in vivo [73]. The study of EVs in bone biology progressed rapidly in the last few years, and more and more papers are emerging on the role of EVs in the determination and regulation of osteoblast pathways [71,74,75]. A key example is the finding that BMSCs-derived exosomes are able to promote osteoblast differentiation by upregulating key osteogenic genes, such as RUNX2, which results in the ability of BMSC-EVs to promote the healing of bone defects in vivo [76,77]. This is at least partially due to the presence of three pro-osteogenic miRNAs in BMSC-EVs, namely miR-196a, miR-27a and miR-206, which were shuttled to osteoblasts and caused the upregulation of the classical osteogenic pathways [76]. A similar report showed that BMSC-EVs shuttle miR-335, which, in turn, targets vesicle-associated membrane protein-associated protein B/C (VapB) in osteoblasts, eventually activating the Wnt/β-catenin pathway and promoting bone fracture healing [77]. Another report showed that the crosstalk went both ways, since mineralizing osteoblasts release EVs that promote the osteogenic differentiation of BMSCs [78], hence it is reasonable to think that an EV-driven feed-forward loop between MSCs and already mature osteoblasts could exist at the actively mineralizing bone surfaces that tend to enhance osteoblast differentiation. M2-macrophages were shown to direct BMSCs toward an osteoblast phenotype through the miRNA-5106/salt-inducible kinase 2/3 axis [79]. Another research group also showed similar results, while demonstrating that M1 macrophages secrete anti-osteogenic EVs, and M0 macrophages-EVs cause similar effects as M2 as well [80]. Urine-derived mesenchymal stem cells (USCs) are functionally similar to BMSCs but, for obvious reasons, their isolation is completely non-invasive, which makes them ideal candidates for autologous cell therapy [81]. Intriguingly, the similarity with BMSCs also held true in the context of EV function in bone biology. In fact, a recent report showed that USC-EVs prevent osteoporosis by enhancing bone formation through the shuttling of collagen triple-helix repeat containing 1 (CTHRC1) to OBs and by reducing osteoclastic bone resorption via osteoprotegerin (OPG) [82].

**Figure 3:**
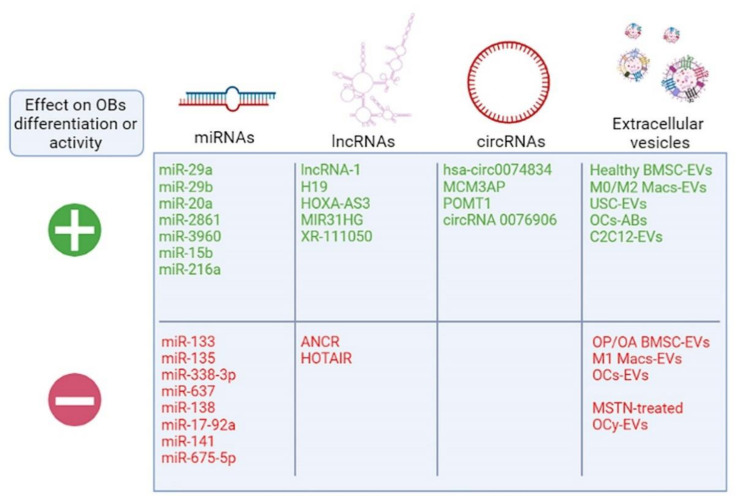
Novel players in osteogenesis. Cartoon listing the non-coding RNAs and extracellular vesicles described in the review. Macs—macrophages; OCs—osteoclasts; OP/OA—osteoporotic/osteoarthritic patients; MSTN—myostatin; OCy—osteocytes. Created with biorender.com.

Speaking of osteoclasts, these are the closest functional partners to the osteoblasts, and their coordinated work in bone remodeling seems to rely also on EVs for their regulation. In fact, osteoclast-EVs are able to specifically shuttle miR-214 to osteoblasts through the interaction between vesicular ephrinA2 and osteoblastic EphA2, which causes a reduction in important osteoblastogenic genes, such as ALP, COL1A1 and OCN [83,84]. Intriguingly, bone remodeling also relies on osteoclasts undergoing apoptosis so that osteoblasts can eventually fill the gap that they created, and this causes the release of apoptotic bodies by mature osteoclasts. These osteoclast-derived apoptotic bodies that are essentially a subset of EVs were shown to promote osteogenic differentiation through receptor activator of nuclear factor κB ligand (RANKL) reverse signaling, i.e., a RANK–RANKL-mediated signaling that takes place in osteoblasts and activates the PI3K/AKT/mechanistic target of the rapamycin (mTOR)/ribosomal protein S6 kinase pathway, eventually leading to osteoblast differentiation [85,86]. However, EVs are not only meant to maintain local homeostasis, and they can be exploited as means to communicate with more distant but functionally associated organs, both directly and indirectly. A close collaborator of bone is certainly muscle, and a small number of reports describing a possible role for EVs in the crosstalk between the two organs have emerged. It was reported that C2C12 myoblasts activate the Wnt/β-Catenin pathway in MC3T3 osteoblasts through EV-shuttled miR-27a-3p [87], while the same EVs were also able to reduce osteoclastogenesis [88,89], thus suggesting that muscle-derived EVs could be a novel mechanism of bone-muscle crosstalk in addition to myokines. Another recent study suggested that myostatin, a myokine that reduces muscle growth, affects osteocytes by promoting their expression of sclerostin (SOST), DKK1 and RANKL [90]. Intriguingly, myostatin also promoted the release of EVs from cultured osteocytes, which were able to target MC3T3 osteoblast precursors and reduce their expression of RUNX2, possibly through the reduction of EV-shuttled miR-218 [90]. Although these reports only show in vitro proof of the mechanism, the participation of EVs in the bone–muscle crosstalk is an emerging topic that could offer important insights and therapeutic targets for both bone and muscle disease prevention [91]. A summary cartoon of the novel players involved in osteogenesis discussed in this review is presented in Figure 3. Importantly, not only do EVs from other cells regulate osteoblast pathways, but also osteoblast-EVs are able to affect other cells. Intriguingly, it was demonstrated that OB-EVs present RANKL on their surface and inside, and they are, therefore, able to promote osteoclast differentiation and survival [92,93].

Given their osteotropism, we also tested whether OB-EVs can be effective drug delivery systems to shuttle anti-resorptive drugs directly into osteoclasts, potentially preventing side effects and increasing the therapeutic index. This was the case for both a bisphosphonate (zoledronic acid) and the proto-oncogene tyrosine-protein kinase Src inhibitor dasatinib [92]. BMSC-EVs were also shown to play a role in angiogenesis. This process has to be closely coupled with osteogenesis, especially during growth; it is, therefore, not surprising that the two processes are also connected through EVs. In fact, a recent paper from Tekeuchi and colleagues confirmed that EVs from BMSCs increase bone regeneration and fracture healing, but proposed that this is through the induction of angiogenesis through EV-shuttled vascular endothelial growth factor (VEGF) [94]. Similar results were shown by Tang and colleagues, who used mature osteoblasts-EVs instead of BMSC-EVs [95]. In our lab, we also noticed that OB-EVs can increase in vivo angiogenesis in physiopathological conditions, i.e., both when using naïve OB-EVs, and even more when using OB-EVs that are isolated from cells previously conditioned with breast cancer-derived factors, hence simulating a bone metastatic milieu [96]. All in all, OB-EVs are extremely versatile and can be an important tool or target for bone metastases as well [96].

## 4. Conclusions

Despite composing a relatively small percentage of the total bone cells (4–5%), osteoblasts are extremely important in the physiopathology of this organ. However, the important role of building the skeleton during growth and maintaining its functionality through the entire life of the individual is not an easy task, and it would not be possible unless a very complex and well-controlled signaling network is in place to control their differentiation and activity. Although the classical differentiation pathways that take MSCs all the way to osteocytes (i.e., Wnt/β-catenin and BMP) are still considered to be most important, today, we know that the situation is much more complex. In fact, researchers have shown that the network controlling expression, stability, post-translational modifications and stability of the key effector proteins identified over the years (i.e., RUNX2, OSX, ALP, COL1A1, Wnt and BMP pathway members) is composed of thousands of other molecules. In this review, we focused on ncRNAs, especially on circRNAs, miRNAs and lncRNAs, all of which have specific features allowing the control the expression and mRNA stability of their target genes. Most of the ncRNAs described so far influence the expression of one or more key osteoblast differentiation genes, which explains their effect in this process. Intriguingly, ncRNAs are often found in abundance in EVs, secreted by virtually all cell types, including atypical ones, such as red blood cells [97]. However, EVs are even more complex and shuttle several other molecules, including proteins, lipids and other nucleic acids, which can also act on cellular metabolism, differentiation and activity. Thus, it should not come as a surprise that more and more reports are emerging that demonstrate a key role of EVs shuttled by other cell types in controlling local osteoblast differentiation. These cells include BMSCs, macrophages, osteoclasts and myoblasts, which all concur to determine the fate of MSCs, and the activity of already differentiated osteoblasts. Furthermore, osteoblasts themselves, which were first thought to only secrete matrix vesicles, were demonstrated by us and other groups to secrete signaling mediating EVs, which can signal to osteoclasts, endothelial cells, BMSCs and cancer cells. EVs are, for their very nature, specific to certain cell types, and, in addition to their well-described use as biomarkers, they hold much promise for drug delivery as well.

In conclusion, while much is known about osteoblast signaling, it seems that we are just scratching the surface, and more research on this topic will allow us to find new targetable pathways and delivery methods for common (e.g., senile or post-menopausal osteoporosis), rare (e.g., osteogenesis imperfecta and cleidocranial dysplasia), and neoplastic (e.g., osteosarcoma and prostate cancer bone metastases) bone diseases.

## Data Availability

Original research papers were found using NIH Pubmed (https://pubmed.ncbi.nlm.nih.gov/ (accessed on April–May 2021)) or google scholar (https://scholar.google.com/ (accessed on April–May 2021)). For searching articles, the keywords used were “osteoblasts” and the relevant factor (e.g., “miRNA” or “extracellular vesicles”). Papers were selected based on impact (number of citations/year), relevance and scientific quality, based on a preliminary abstract screening and a further in-depth critical read of the selected articles. Some of the articles were already present in the authors’ libraries and were included where appropriate.
